# Combined Vacuum and Ascorbic Acid Treatment Enhances Texture and Antioxidant Capacity in Fresh-Cut Potatoes: Transcriptomic Elucidation of Glutathione Metabolism Mechanisms

**DOI:** 10.3390/foods15010035

**Published:** 2025-12-22

**Authors:** Ronghui Fang, Xinyi Wei, Qi Qu, Pingfan Rao, Shutao Liu

**Affiliations:** 1 Zhicheng College, Fuzhou University, Fuzhou 350002, China; 02119844@fdzcxy.edu.cn; 2 Institute of Biotechnology, Fuzhou University, Fuzhou 350002, China; 3 Haixia Institute of Science and Technology, Fujian Agriculture and Forestry University, Fuzhou 350002, China

**Keywords:** potato, vacuum packaging, ascorbic acid, transcriptome, texture, antioxidant capacity, glutathione metabolism

## Abstract

This study investigated the mechanism by which combined vacuum packaging and 0.5% (*w*/*v*) ascorbic acid treatment (VP-AsA) preserves fresh-cut potatoes at 4 °C, integrating physiological and transcriptomic analyses. Transcriptome sequencing revealed 2246 differentially expressed genes (DEGs) in the VP-AsA group. Notably, key genes involved in glutathione metabolism and NADPH regeneration—encoding glutathione reductase (GR), glutathione S-transferase (GST), isocitrate dehydrogenase (IDH), glucose-6-phosphate dehydrogenase (G6PDH), and ornithine decarboxylase (ODC)—were significantly up-regulated. This transcriptional reprogramming, which was associated with increased glutathione (GSH) content, provides a molecular basis for the enhanced antioxidant capacity observed in the treated samples, including elevated superoxide dismutase (SOD) activity, DPPH/ABTS radical scavenging capacity, and ferric reducing antioxidant power (FRAP). Concurrently, VP-AsA treatment reduced water migration, inhibited polyphenol oxidase (PPO) and peroxidase (POD) activities, and maintained key textural properties (hardness, fracturability, springiness, chewiness) during the first 9 days of storage. These results suggest that VP-AsA treatment preserves quality at least in part by transcriptionally activating glutathione-mediated antioxidant pathways, providing insights for fresh-cut fruits and vegetables quality control.

## 1. Introduction

The potato (*Solanum tuberosum* L.), a member of the Solanaceae family, is the world’s fourth most important food crop following wheat, rice, and maize [1]. Economic development and evolving consumer preferences have driven the popularity of fresh-cut foods, with fresh-cut potato being a prominent example. However, the cutting process damages the cellular integrity of produce, leading to moisture and nutrient loss during storage. This subsequently induces quality deterioration, manifested as texture softening, browning and other deteriorations [2]. In fresh-cut potatoes, water redistribution—driven by the migration of bound water to free water, modifications in cell wall composition, and starch hydrolysis—adversely affects the product’s textural properties.

In addition to water loss, oxidative browning and reactive oxygen species (ROS) accumulation are major factors contributing to quality decline in fresh-cut potatoes. To delay quality deterioration, the combined treatment of vacuum packaging (VP) and ascorbic acid (AsA), denoted as VP-AsA, has shown promising potential. Previous studies have demonstrated that vacuum packaging significantly reduces the oxygen partial pressure, thereby slowing the polyphenol oxidase (PPO)-catalyzed reaction and delaying enzymatic browning [3]. AsA, a safe and efficient reducing agent [4,5], effectively inhibits browning reactions in various fruits and vegetables (e.g., lettuce [6], purple cabbage [7], banana [8]), enhances antioxidant and ROS-scavenging capacities [9,10], and regulates respiration and ethylene metabolism. Zhou et al. reported that exogenous AsA application suppresses wound healing in fresh-cut potatoes [11]. Recent studies indicate that combined preservation methods can significantly improve the quality and extend the shelf life of fresh-cut produce. Our previous study [12] established the synergistic effect of combining vacuum packaging with 0.5% (*w*/*v*) ascorbic acid (VP-AsA), a conclusion derived from a systematic screening that included the evaluation of individual AsA concentrations, packaging photographs (VP-AsA), and a comparison of vacuum versus atmospheric packaging without ascorbic acid. Therefore, this study investigates the textural and antioxidative physiological mechanisms underlying this synergy.

Transcriptome sequencing (RNA-seq) provides a comprehensive and quantitative overview of gene expression dynamics and has become a powerful tool for elucidating molecular regulatory mechanisms in postharvest physiology [13]. In horticultural products, RNA-seq has revealed key pathways associated with antioxidant defense, membrane integrity, phenylpropanoid metabolism, and signal transduction, linking transcriptional regulation to phenotypes such as browning intensity, respiration rate, membrane permeability, and malondialdehyde (MDA) accumulation [9,13,14]. Beyond these whole-fruit studies, RNA-seq has also been applied to fresh-cut systems. For instance, a recent transcriptomic analysis of fresh-cut eggplant identified 8347 differentially expressed genes and highlighted PPO, POD, PAL, CAT and other oxidation-related gene families as central drivers of enzymatic browning, providing direct molecular evidence for cut-surface browning mechanisms in minimally processed produce [15]. Complementary work in litchi demonstrated that preservative treatment modulates oxidation–reduction processes and suppresses phenylpropanoid biosynthesis, thereby reducing enzymatic browning and decay [16], while studies in pineapple have shown that exogenous AsA treatment enhances antioxidant gene expression and maintains glutathione (GSH) homeostasis to delay black heart development [17]. These studies illustrate the power of transcriptomics in elucidating preservation mechanisms in various horticultural products.

However, despite these advances, the transcriptional regulatory mechanisms through which the combined VP-AsA treatment modulates gene expression—particularly within glutathione metabolism and antioxidant defense pathways—remain to be systematically elucidated in specifically fresh-cut potatoes. To address this gap, the present study employs an integrated multi-omics strategy. We combine physiological analyses (water status, texture, antioxidant capacity) with transcriptome sequencing to decipher how VP-AsA treatment transcriptionally reprograms metabolic pathways to enhance preservation. This work specifically aims to elucidate the gene-level regulatory networks underlying the observed synergy, with a focus on glutathione-mediated antioxidant pathways, thereby bridging the gap between physiological effects and their molecular determinants.

## 2. Materials and Methods

### 2.1. Materials and Reagents

Fresh potato tubers (*Solanum tuberosum* L. cv. Holland No. 15) were procured from Qingdao Jinmandi Seed Industry Co., Ltd. (Tengzhou, China). To ensure uniformity and consistency of the experimental materials, all tubers were sourced from the same field plot and harvested on the same day. Although specific biochemical indices for potato maturity at harvest were not measured, visual inspection and strict selection criteria were applied. Specifically, tubers exhibiting uniform size, regular shape, intact skin without greening, sprouting, or mechanical injuries were meticulously selected for the experiments. Holland No. 15 was selected because it is widely cultivated and exhibits stable tuber quality. After harvest, the tubers were transported to the laboratory in cardboard boxes lined with polyethylene foam and ice packs. Tubers exhibiting no signs of sprouting, mildew, black spots, softening, or greening were meticulously selected and stored at (4 ± 0.5) °C in a Haier BCD-571WDPF refrigerator for no more than 24 h (humidity was not strictly controlled as processing was completed within 24 h) pending subsequent experiments. Vacuum packaging bags, fabricated from nylon seven-layer co-extruded film (oxygen transmission rate, OTR: 40 cm^3^/m^2^·24 h·atm) and measuring 12 × 17 cm, were obtained from Dongguang Xinxin Plastic Co., Ltd. (Dongguang, China). The vacuum level applied during packaging was 500 mm Hg (approximately 66.5 kPa). Food-grade ascorbic acid was supplied by Heilongjiang Xinhecheng Biotechnology Co., Ltd. (Harbin, China). The following reagents were purchased from respective manufacturers: 1,1-diphenyl-2-picrylhydrazyl (DPPH, ≥98% purity) from Shanghai Yuanye Bio-Technology Co., Ltd. (Shanghai, China); polyvinylpolypyrrolidone (PVPP, ≥99% purity) from Anhui Youpin Biological Technology Co., Ltd. (Hefei, China); 2,2′-azino-bis(3-ethylbenzothiazoline-6-sulfonic acid) (ABTS, ≥98% purity), 2,4,6-tripyridyl-s-triazine (TPTZ, ≥98% purity), and dithiothreitol (DTT, ≥98% purity) from Beijing Solarbio Science & Technology Co., Ltd. (Beijing, China). A commercial Reduced Glutathione Assay Kit (model A006-1-1, Nanjing Jiancheng Bioengineering Institute, Nanjing, China).

### 2.2. Instruments and Equipment

The following instruments were used in this study: a YP-1002 electronic analytical balance (Sartorius AG, Göttingen, Germany); a BCD-571WDPF refrigerator (Haier Co., Ltd., Qingdao, China); a TA-XT Plus texture analyzer (Stable Micro Systems Ltd., Godalming, UK; distributed by Beijing Microsignal Advanced Instrument Technology Co., Ltd., Beijing, China); an NMI20-060H-I nuclear magnetic resonance (NMR) imaging analyzer (Niumag Corporation, Suzhou, China); a SpectraMax ID3 multifunctional microplate reader (Molecular Devices LLC., San Jose, CA, USA) and a CARY50 UV-visible spectrophotometer (Agilent Technologies, Santa Clara, CA, USA); a DZ-400/2D Single-Chamber Vacuum Packaging Machine (Changzhou Kaili Machinery Co., Ltd., Changzhou, China); and a CF15RX high-speed refrigerated centrifuge (Hitachi, Tokyo, Japan).

### 2.3. Experimental Methods

#### 2.3.1. Preparation of Fresh-Cut Potato Shreds

Fresh potatoes were removed from 4 °C storage and equilibrated at 20 °C in darkness for 24 h. The tubers were washed with tap water and surface-disinfected by immersion in a 0.02% (*v*/*v*) sodium hypochlorite (NaClO) solution for 5 min. After disinfection, the potatoes were blotted dry with sterile absorbent paper. All tools and utensils were sanitized by immersion in the same NaClO solution (5 min) followed by thorough rinsing with deionized water.

The potatoes were manually peeled and cut into uniform shreds (approximately 3 mm × 3 mm in cross-section) using a sharp knife. The shreds were immediately rinsed with deionized water to remove surface starch. These surface-starch-washing shreds, derived from a total of 9 potato tubers, were first thoroughly homogenized to form a uniform pool (eliminating individual tuber variability) and then randomly divided into two groups. One group was soaked in deionized water for 5 min, while the other was soaked in 0.5% (*w*/*v*) ascorbic acid solution for 5 min. After draining, samples were spun in a centrifuge for 10 s to remove residual moisture. Subsequently the fresh-cut potatoes prepared from multiple tubers were thoroughly homogenized before packaging. Each sample (30 ± 1 g) was vacuum-packaged and assigned to one of two treatments: the control group (CK, vacuum packaging alone) or the combined treatment group (VP-AsA). All packages were stored at 4 °C. The internal relative humidity within the vacuum-sealed bags was not monitored throughout the storage period. This is because the nylon seven-layer co-extruded film used provides a high barrier against moisture, which inherently maintains a stable, high-humidity microenvironment around the samples, preventing significant moisture loss.

Samples were collected on day 0, 3, 6, 9, 12, and 15 of storage:

Texture/Water Analysis: 1 g samples were randomly collected from 3 random positions within each packaging unit (3 biological replicates/treatment).

Biochemical Assays: 10 g composite samples (each pooled from 3 random positions within individual biological replicates) were snap-frozen in liquid nitrogen and stored at −80 °C.

#### 2.3.2. Texture Profile Analysis of Fresh-Cut Potatoes During Storage

Texture profile analysis (TPA) was performed using a texture analyzer according to the method described by Hunjek et al. [18] with slight modifications. The TPA test was conducted using a P/36R cylindrical probe. The testing conditions were set as follows: pre-test speed, 2.0 mm/s; test speed, 2.0 mm/s; post-test speed, 2.0 mm/s; compression level, 90%; trigger force, 5.0 g; and a total test time of 5.00 s. The probe compressed the potato shreds twice to achieve the specified deformation. For each condition, measurements were performed in nine replicates. Based on the parameters output by the instrument’s software, hardness (g), fracturability (g), springiness, and chewiness were selected from the TPA to analyze the textural changes in the CK and VP-AsA groups of fresh-cut potatoes over 15 days of storage.

#### 2.3.3. Determination of Water Status in Fresh-Cut Potato Shreds

LF-NMR has been widely applied to characterize the physical and biological properties of water in biological tissues [19]. The water status of fresh-cut potato shreds during storage was determined according to the method described by Wang et al. [20] with slight modifications. Transverse relaxation time (T_2_) was measured using a low-field nuclear magnetic resonance (LF-NMR) analyzer (Niumag Corporation, Suzhou, China), employing the Carr-Purcell-Meiboom-Gill (CPMG) pulse sequence. Magnetic resonance imaging (MRI) was simultaneously performed to analyze hydrogen proton density images, thereby evaluating the water distribution within the samples during storage, referencing the method of Wang [21] with modifications.

For each condition, 1 g of sample was analyzed in triplicate using the CPMG sequence with the following parameters: sampling frequency (SW), 200 kHz; wait time (TW), 3500 ms; echo time (TE), 0.8 ms; number of echoes (NECH), 10,000; number of scans (NS), 4; analog gain (RG1), 20 dB; and digital gain (DRG1), 3.

For MRI analysis, 3 g samples were used under each condition with repetition time (TR) 500 ms, echo time (TE) 13.44 ms, field of view read (FOV Read) 120 mm, field of view phase (FOV Phase) 115 mm, and slice thickness 10 mm.

The acquired proton density images were processed using Niumag NMR image processing software (V3.0, Niumag Corporation, Suzhou, China). To enable direct visual comparison across storage days, all MRI images were normalized to a uniform intensity scale using histogram matching algorithms implemented in the software. The images were pseudo-colored using the Hotmetal mode for enhanced visualization and ultimately saved in standard PNG format.

#### 2.3.4. Assay of Polyphenol Oxidase and Peroxidase Activities

The activities of polyphenol oxidase (PPO) and peroxidase (POD) were determined according to the methods described by Wei et al. [22] and Yan et al. [23], respectively, with slight modifications.

Enzyme extraction: Approximately 1 g of potato tissue and 0.12 g of polyvinylpolypyrrolidone (PVPP) were homogenized in a centrifuge tube with 5 mL of ice-cold phosphate buffer (0.1 mol/L, pH 6.8). The homogenate was vortexed thoroughly and kept on ice. The mixture was then centrifuged at 8000× *g* and 4 °C for 15 min. The resulting supernatant was collected and used as the crude enzyme extract for subsequent assays.

PPO activity assay: The reaction mixture consisted of 1 mL of 50 mM catechol, 1.5 mL of phosphate buffer, and 0.5 mL of the crude enzyme extract. The mixture was rapidly vortexed, and the increase in absorbance at 420 nm was immediately recorded at 30 s intervals for 3 min.

POD activity assay: For the POD assay, 0.5 mL of the crude enzyme extract was mixed with 3 mL of 25 mM guaiacol solution, followed by the addition of 50 mM H_2_O_2_ solution to initiate the reaction. The mixture was rapidly vortexed, and the increase in absorbance at 470 nm was recorded immediately at 30 s intervals for 3 min.

For both enzymes, one unit of enzyme activity (U) was defined as an increase of 0.01 in absorbance per minute. The enzyme activity was expressed in units per gram of fresh weight (U/g) and calculated using the following Formula (1):

Enzyme activity (U/g) = (ΔA × V_T_)/(m × V_S_× t × 0.01) (1)

where ΔA represents the change in absorbance at the respective wavelength (420 nm for PPO and 470 nm for POD), VT is the total volume of the extraction buffer (mL), vs. is the volume of the crude enzyme extract used in the assay (mL), t is the reaction time (min), and m is the fresh weight of the sample (g). Enzyme activity is expressed in units (U), where one unit corresponds to an absorbance change of 0.01 per minute.

#### 2.3.5. Determination of Superoxide Dismutase Activity and Antioxidant Capacity

##### Superoxide Dismutase (SOD) Activity Assay

SOD activity was determined according to the method of Lin et al. [24] with modifications. Fresh potato tissue (1 g) was homogenized in 5 mL of ice-cold 0.1 M phosphate extraction buffer (pH 7.8) containing 5% (*w*/*v*) PVPP and 5 mM DTT. The homogenate was centrifuged at 10,000× *g* and 4 °C for 15 min, and the supernatant was collected for analysis. The reaction mixture consisted of 1.3 mL of 50 mM phosphate buffer (pH 7.8), 0.3 mL of 130 mM methionine, 0.3 mL of 750 μM NBT, 0.3 mL of 100 μM EDTA-Na_2_, 0.3 mL of 20 μM riboflavin, and 0.1 mL of enzyme extract. After 20 min of illumination (4000 Lux), the reaction was terminated by transferring the tubes to darkness. Absorbance was measured at 560 nm. One unit of SOD activity (U) was defined as the amount of enzyme inhibiting 50% of NBT reduction per minute per gram of fresh tissue. Results were expressed as U/g FW.

##### Antioxidant Capacity Assay

Antioxidant capacity was evaluated using DPPH, ABTS, and FRAP assays based on methods by Baek [25] and Choi et al. [26] with modifications. For sample preparation, 1 g of tissue was extracted with 5 mL of 95% ethanol under ultrasonication at 25 °C for 30 min, followed by centrifugation at 8000× *g* and 4 °C for 15 min.

For the DPPH assay, 0.2 mL of sample extract was mixed with 0.8 mL of 0.4 mM DPPH solution, incubated in darkness for 10 min, and measured at 517 nm.

For the ABTS assay, 100 μL of sample was reacted with 1 mL of ABTS^+^ solution for 30 min in darkness, and absorbance was recorded at 734 nm.

For the FRAP assay, 0.05 mL of sample was combined with 0.15 mL distilled water and 1.5 mL FRAP working solution, incubated at 37 °C for 10 min, and measured at 595 nm.

Radical scavenging activity was calculated as:

(2) $$Scavenging\;activity\left(\%\right)=\;\left[1-\frac{A_{1}}{A_{0}}\right]\times 100\%$$

where *A*_1_ represents the absorbance of the sample, and *A*_0_ represents the absorbance of the blank control.

#### 2.3.6. Determination of Reduced Glutathione (GSH) Content

The reduced glutathione (GSH) content in fresh-cut potato tissue samples was quantified using a commercial Reduced Glutathione Assay Kit, following the manufacturer’s instructions. The GSH concentration was calculated based on the standard curve obtained from the provided standards.

#### 2.3.7. RNA Extraction and Quality Control

Each treatment group (CK and VP-AsA) had three independent biological replicates. On day 9, two of these three replicates were selected for transcriptome analysis, and the corresponding samples (2 g each) were sent to Beijing Ovison Genomics for RNA extraction. Day 9 was selected because it marks a key transition when the control (CK) exhibited significant declines in hardness and fracturability, while the VP-AsA treatment maintained stability (as presented in Section 3.3).

Total RNA was isolated using the TRIzol reagent method, followed by treatment with RNase-free DNase I (Takara, Kusatsu, Japan) to remove genomic DNA contamination. RNA integrity and potential contamination were assessed by electrophoresis on a 1% (*w*/*v*) agarose gel. RNA concentration and purity were measured using a NanoDrop spectrophotometer (Thermo Fisher Scientific, Waltham, MA, USA), while RNA integrity was evaluated with an Agilent 2100 Bioanalyzer (Agilent Technologies, Santa Clara, CA, USA).

#### 2.3.8. Transcriptome Sequencing Library Construction

Sequencing libraries were prepared using the NEBNext Ultra RNA Library Prep Kit (New England Biolabs, Ipswich, MA, USA) according to the manufacturer’s protocol. Briefly, 1.5 μg of total RNA was used for library construction. mRNA was enriched using poly-T magnetic beads and fragmented under elevated temperature in the presence of divalent cations. First-strand cDNA was synthesized using random hexamer primers and M-MuLV Reverse Transcriptase, followed by second-strand cDNA synthesis using DNA Polymerase I and RNase H. After end repair, adenylation, and ligation of NEBNext hairpin loop adapters, cDNA fragments of 200–250 bp were selected using AMPure XP beads (Beckman Coulter, Indianapolis, IN, USA).

PCR amplification of the adapter-ligated DNA libraries was performed in a 50 μL reaction, consisting of 25 μL 2 × Phusion High-Fidelity PCR Master Mix (NEB), 5 μL Universal PCR Primer, 5 μL Index Primer, and 15 μL purified, adaptor-ligated cDNA. Amplification was carried out using a Bio-Rad T100 thermal cycler with the following conditions: an initial denaturation at 98 °C for 30 s, followed by 12 cycles of 98 °C for 10 s, 65 °C for 30 s, and 72 °C for 30 s, with a final extension at 72 °C for 5 min. The number of PCR cycles was selected based on NEB recommendations to ensure sufficient amplification while avoiding over-amplification.

Library quality was assessed using an Agilent 2100 Bioanalyzer (Agilent Technologies, Santa Clara, CA, USA) to verify fragment size distribution and detect adapter dimers. Library concentration was quantified using a Qubit 3.0 fluorometer (Thermo Fisher Scientific) to ensure accurate pooling prior to sequencing.

The purified cDNA libraries were sequenced on an Illumina NovaSeq 6000 platform by Beijing Allwegene Technology Company Limited (Beijing, China) using a paired-end 150 bp (PE150) strategy. For each sample, approximately 5.8–6.4 Gb of sequencing data were generated, corresponding to about 39–43 million raw reads per library.

#### 2.3.9. Data Quality Control and Alignment

To ensure data reliability, raw sequencing reads were processed using fastp (v0.20.1) [27] to remove low-quality reads, adapter sequences, and reads with abnormal GC content distribution, yielding clean reads for downstream analysis. At the same time, Q20, Q30, GC-content and sequence duplication level of the clean data were calculated (see Results for details).

Fresh-cut samples originated from the potato cultivar “Holland No. 15” and Clean reads were aligned to the Solanum tuberosum reference genome (version 4.03). The genome is downloaded from the Phytozome database (https://phytozome-next.jgi.doe.gov/info/Stuberosum_v4_03 (accessed on 16 January 2023)). Reads alignment was conducted using STAR (v2.7.11a) [28] with the following major parameters: “—quantMode TranscriptomeSAM GeneCounts—outFilterMismatchNmax 10—alignIntronMax 500,000—alignIntronMin 20—outSAMtype BAM SortedByCoordinate—outFilterMismatchNmax 2”. These parameters ensure accurate mapping of reads to the genome, and the overall alignment rate for all samples ranged from 95.06% to 95.89%.

#### 2.3.10. Differential Expression and Enrichment Analysis

Differentially expressed genes (DEGs) were identified using the DESeq2 package (v1.44.0) [29] with the following threshold criteria: absolute log_2_ fold change (|log_2_FC|) > 2 and adjusted *p*-value (padj) < 0.05. The original PGSC gene IDs of the DEGs were converted to ENTREZ IDs using the AnnotationHub R package (v3.8.0), selecting the AH114724 potato annotation library. Gene Ontology (GO) and Kyoto Encyclopedia of Genes and Genomes (KEGG) enrichment analyses were performed separately on both up-regulated and down-regulated DEGs using the clusterProfiler R package (v4.12.6) [30]. The results were visualized using ggplot2 (v.3.4.2). Enriched KEGG pathway diagrams were generated using the Pathview R package (v.1.40.0), which maps gene expression data noto KEGG native pathway diagrams based on curated pathway information.

### 2.4. Statistical Analyses

Physicochemical and texture analyses were performed with three independent biological replicates (*n* = 3) per treatment group. For transcriptomic analysis, one sample was taken from two of the three independent replicates on day 9 for RNA sequencing. Experimental data were analyzed using SPSS 27.0 (IBM Corp., Armonk, NY, USA) for two—way ANOVA and principal component analysis (PCA). Statistical significance (α = *p* < 0.05) was determined by Tukey’s post—hoc test for multiple comparisons. The *p*-value ranges were reported as * *p* < 0.05, ** *p* < 0.01, and *** *p* < 0.001; in the graphical representations (generated via GraphPad Prism 10.1.1, GraphPad Software, San Diego, CA, USA), these symbols denote the levels of statistical difference between compared groups. Results are expressed as mean ± SD.

## 3. Results and Analysis

### 3.1. Water Migration and Distribution

**Figure 1:**
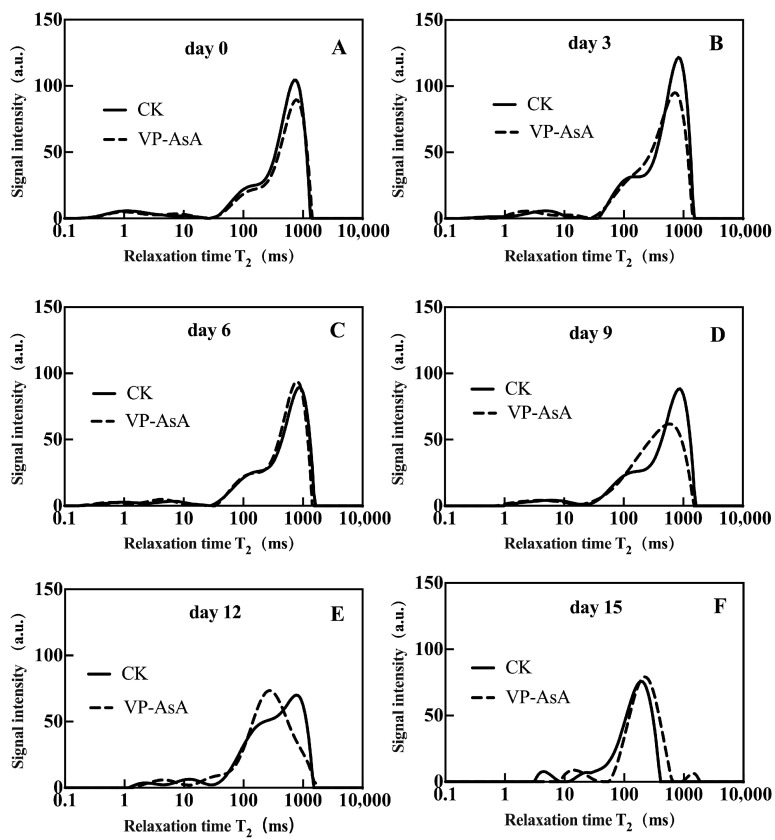
Distribution of lateral relaxation time (T2) during storage of fresh-cut potatoes. The T_2_ relaxation spectra of the control (CK, solid line) and VP-AsA-treated (dashed line) groups are shown. The distributions illustrate the dynamics of water mobility and distribution across different phases. Panels (**A**–**F**) represent the storage days 0, 3, 6, 9, 12, and 15, respectively.

The changes in water status of fresh-cut potatoes during storage were monitored using Low-Field NMR. Figure 1 shows the T_2_ inversion spectra of fresh-cut potatoes over a 15-day storage period. A shorter relaxation time (T_2_), indicated by a peak positioned further to the left, corresponds to protons in a more restricted molecular environment [26]. The peaks are categorized into three distinct water populations based on their T_2_ ranges [31]: T_21_ (0.01–10 ms), representing tightly bound intracellular water [32]; T_22_ (10–200 ms), signifying less mobile water [33]; and T_23_ (200–1000 ms), corresponding to free water. The dynamics of the bound (T_21_, T_22_) and free (T_23_) water fractions are analyzed below.

**Figure 2:**
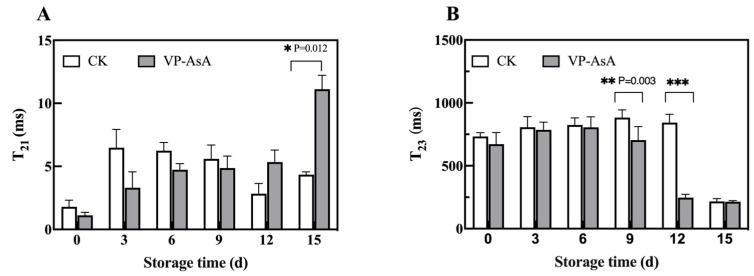
Changes in relaxation time T_21_ and T_23_ parameters of fresh-cut potatoes during storage. Panel (**A**) shows the T_21_ values of the control (CK) and VP-AsA treatment groups at different storage times (0–15 days); Panel (**B**) shows the corresponding T_23_ values. *, **, and *** Indicate significant differences between the control (CK) and VP-AsA treatment groups at *p* < 0.05, *p* < 0.01, and *p* < 0.001, respectively.

Analysis of the relaxation times for bound water (T_21_) and free water (T_23_) provided quantitative insights into water migration. Figure 2A presents the changes in the relaxation time (T_21_) of bound water during storage. No significant differences were observed between the two treatment groups in the early storage period (0–12 days), while a significant (*p* < 0.05) difference emerged only on day 15. As shown in Figure 2B, the relaxation time (T_23_) of free water exhibited no significant differences between the two groups during the initial storage phase (0–6 days). However, the VP-AsA group showed significantly (*p* = 0.003 on day 9; *p* < 0.001 on day 12) lower T_23_ values than the CK group on days 9 and 12. Combined with data from Table S1, the bound water content (A_21_) in CK (3.45%) was markedly depressed relative to VP-AsA (5.01%) on day 15. These findings demonstrate that VP-AsA treatment effectively delayed water migration during storage and contributed to maintaining a stable cellular environment in fresh-cut potatoes.

### 3.2. MRI Image Changes During Storage of Fresh-Cut Potatoes

**Figure 3:**
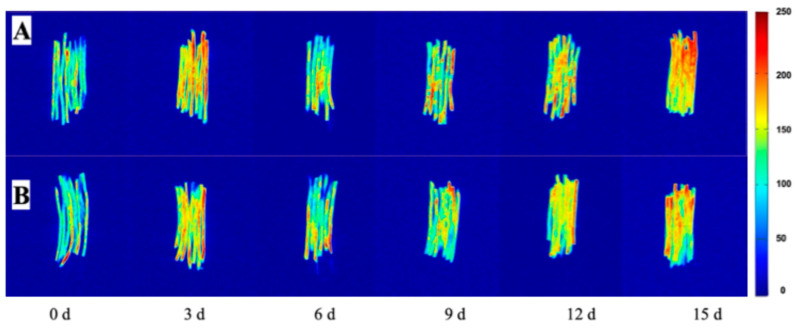
Changes in MRI images of fresh-cut potatoes during storage. Note: (**A**) Control group (CK); (**B**) VP-AsA treatment group.

To visually demonstrate the changes in spatial distribution of water molecules, we conducted observations using magnetic resonance imaging (MRI). Figure 3 illustrates the spatial distribution of water protons, with the color gradient from red to blue representing decreasing proton density levels. A progressive increase in water proton density was observed in fresh-cut potatoes throughout the storage period. Beginning at day 9, the control (CK) group exhibited more intense red coloration, indicating localized areas of elevated proton density. This shift suggests that water molecules within the CK samples had undergone significant migration, with an increasing proportion transitioning into the free water state, which is a direct visual manifestation of microstructural breakdown and declining water-holding capacity at the cellular level. In contrast, the VP-AsA treatment group maintained a more stable water distribution throughout the storage period, with the appearance of intense red areas being significantly delayed and less extensive, demonstrating that the treatment effectively retarded water migration and the formation of free water.

### 3.3. Texture Profile Analysis of Fresh-Cut Potatoes

**Figure 4:**
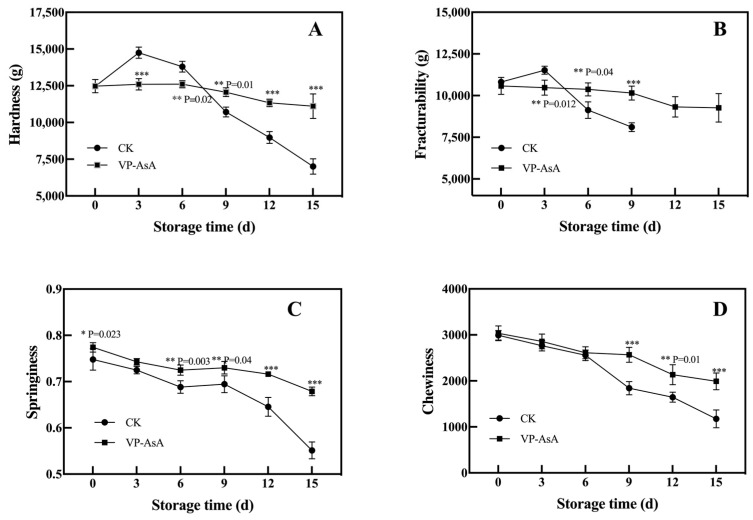
Texture changes in fresh-cut potatoes during storage. (**A**) Hardness, (**B**) Fracturability, (**C**) Springiness, (**D**) Chewiness. The changes in each parameter for the control (CK) and VP-AsA treatment groups at different storage times are shown. *, **, and *** indicate significant differences between the control (CK) and VP-AsA treatment groups at *p* < 0.05, *p* < 0.01, and *p* < 0.001, respectively.

Texture profile analysis demonstrated that VP-AsA treatment effectively maintained the textural quality of fresh-cut potatoes during storage (Figure 4). As shown in Figure 4A, the VP-AsA treatment significantly (*p* = 0.01) delayed the decrease in hardness after day 9 of storage. For fracturability, VP-AsA treatment significantly extended its detectable retention time to 15 days, whereas it became undetectable in the CK group after 9 days. Meanwhile, the VP-AsA group exhibited significantly higher springiness (*p* < 0.05) than the CK group throughout the entire storage period. By the end of storage, the reduction in chewiness was significantly (*p* < 0.05) smaller in the VP-AsA group compared to the CK group. These results collectively indicate that VP-AsA treatment effectively delays textural deterioration and better preserves the sensory quality of the product.

### 3.4. Impact on PPO and POD Activities

**Figure 5:**
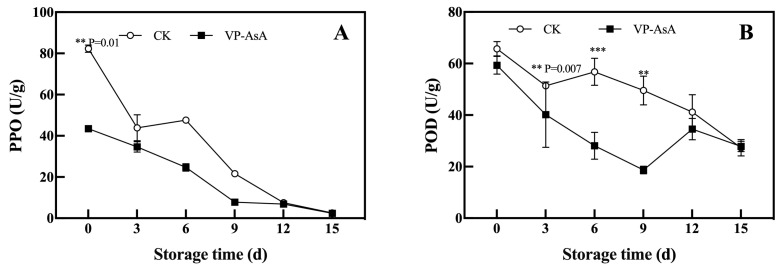
Effect of vacuum packaging combined with ascorbic acid on PPO (**A**) and POD (**B**) during storage of fresh-cut potatoes. **, and *** indicate significant differences between the control (CK) and VP-AsA treatment groups at *p* < 0.01, and *p* < 0.001, respectively.

The activity changes in browning-related enzymes (polyphenol oxidase, PPO, and peroxidase, POD) serve as a critical indicator for evaluating preservation efficacy. Figure 5A shows that PPO activity generally decreased with storage time. The VP-AsA group consistently exhibited lower PPO activity than the control (CK), and the difference was significant (*p* = 0.01) at day 0. Although this difference diminished as activities neared zero by day 15, VP-AsA treatment effectively suppressed initial PPO activity and slowed its progression, thereby reducing phenolic oxidation and browning while preserving product color.

As shown in Figure 5B, VP-AsA treatment significantly inhibited POD activity, particularly during early storage (days 6 and 9), where the difference from CK was highly significant (*p* < 0.001).

### 3.5. Analysis of Superoxide Dismutase Activity

**Figure 6:**
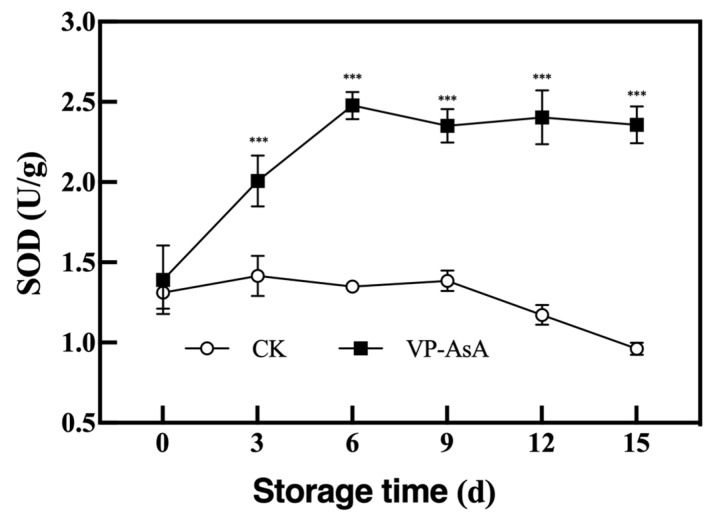
Effect of vacuum packaging combined with ascorbic acid on SOD during storage of fresh-cut potatoes. *** indicate significant differences between the control (CK) and VP-AsA treatment groups at *p* < 0.001.

SOD activity showed an initial increase followed by a decrease during storage, a trend consistent with previous findings reported by Li et al. [34]. From day 3 onwards, the VP-AsA group maintained significantly higher enzymatic activity than the CK group (*p* < 0.001), indicating that the VP-AsA treatment effectively maintained and promoted SOD activity in fresh-cut potatoes (Figure 6).

### 3.6. Determination of Antioxidant Capacity

**Figure 7:**
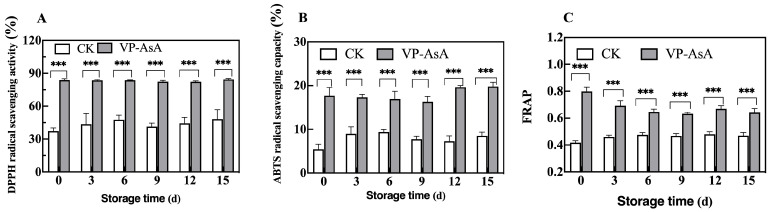
Effect of vacuum packaging combined with ascorbic acid on antioxidant activity of fresh-cut potatoes during storage. (**A**) DPPH radical scavenging activity, (**B**) ABTS radical scavenging capacity, (**C**) FRAP value. The changes in each parameter for the control (CK) and VP-AsA treatment groups at different storage times are shown. *** Indicates significant differences between the control (CK) and VP-AsA treatment groups at *p* < 0.001.

The antioxidant capacity of fresh-cut potatoes was assessed by measuring the DPPH radical scavenging rate, ABTS radical scavenging capacity, and FRAP values. As shown in Figure 7, the VP-AsA group maintained consistently higher values across all three assays compared to the CK group throughout the 15-day storage period. Statistical analysis revealed that the time × treatment interaction (Tables S2–S4) was highly significant for ABTS scavenging capacity (*p* = 0.003) and FRAP values (*p* = 0.000), but not significant for DPPH scavenging rate. The non-significant interaction for DPPH indicates that the enhancing effect of VP-AsA treatment remained relatively stable over time. More importantly, the main effect of “Treatment” was highly significant (*p* < 0.001) across all three assays. This clearly demonstrates that, when the effect of storage time is accounted for, the VP-AsA treatment itself was the primary cause of the differences in antioxidant capacity. These results demonstrate that ascorbic acid treatment effectively enhances the antioxidant activity of fresh-cut potatoes.

### 3.7. Reduced Glutathione (GSH) Content

**Figure 8:**
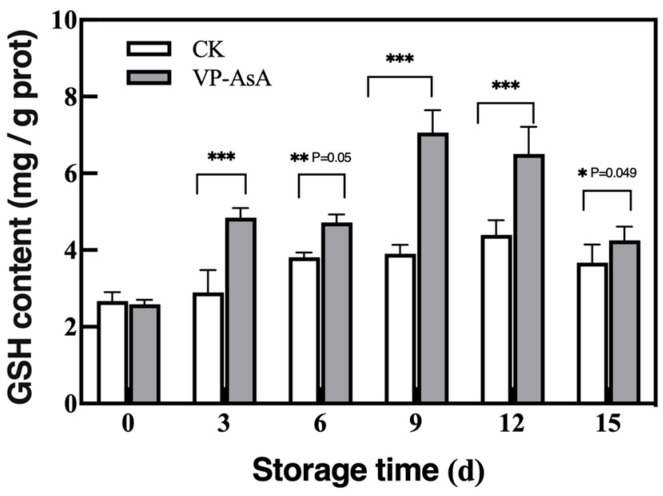
Effect of vacuum packaging synergistic ascorbic acid on reduced glutathione (GSH) content during storage of fresh-cut potatoes. *, **, and *** indicate significant differences between the control (CK) and VP-AsA treatment groups at *p* < 0.05, *p* < 0.01, and *p* < 0.001, respectively.

The reduced glutathione (GSH) content, a key antioxidant in the ascorbate-glutathione cycle, was monitored to assess the treatment’s impact on the antioxidant system. As shown in Figure 8, the GSH content in fresh-cut potatoes under both VP-AsA and CK treatments first increased and then decreased during storage. The VP-AsA group showed higher GSH content than the CK group after day 3. The differences were highly significant (*p* < 0.001) on days 3, 9, and 12, and significant (*p* < 0.05) on day 15. These results indicate that AsA promotes GSH accumulation, thereby enhancing antioxidant activity. The decrease in GSH content in both groups by day 15 may be related to severe quality deterioration in the late storage stage.

### 3.8. Transcriptome Differential Analysis of Fresh-Cut Potatoes

**Figure 9:**
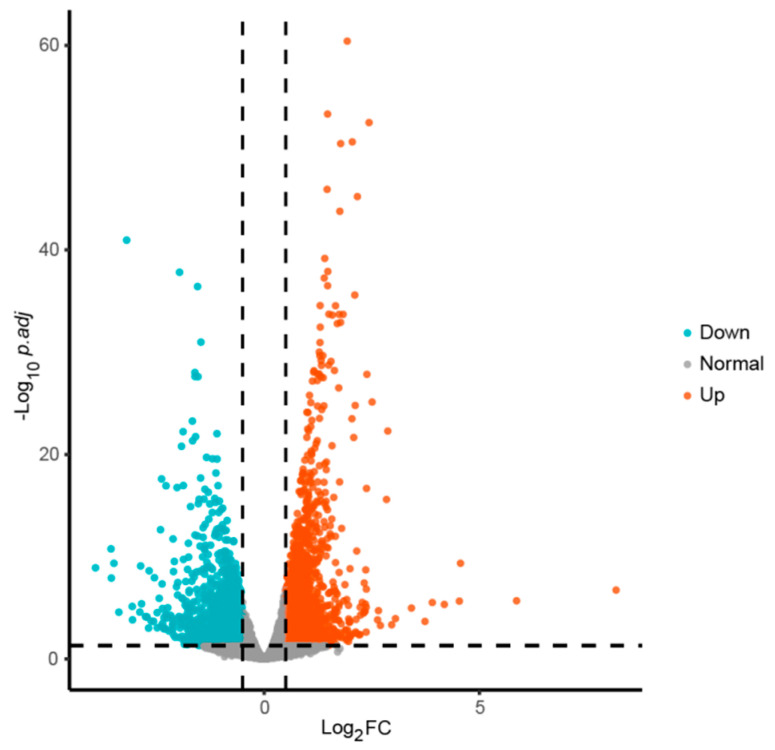
Volcano plot of differentially expressed genes at day 9 during fresh-cut potato storage. The x-axis represents log2 fold change (log2FC), and the y-axis represents −log10 adjusted *p*-values (−log10 *p.adj*). The vertical dashed lines indicate the thresholds for fold change, and the horizontal dashed line indicates the significance cutoff. Genes significantly upregulated are shown in orange, whereas significantly downregulated genes are shown in blue-green.

To elucidate the molecular basis for the enhanced antioxidant activity, transcriptome analysis compared VP-AsA-treated fresh-cut potatoes (vacuum packaging with 0.5% (*w*/*v*) ascorbic acid) against vacuum-packed controls (CK) after 9 days of storage. High-quality RNA-seq datasets were obtained for all samples, with each library generating 39–43 million raw reads (5.8–6.4 Gb per sample). After filtering, the number of clean reads remained nearly identical to raw reads, and the sequencing error rate was consistently 0.03%. The base quality was also high, with Q20 values of 97.56–97.91%, Q30 values of 93.00–93.83%, and GC content ranging from 42.63% to 42.70%. These metrics have confirmed the reliability of the datasets for downstream transcriptome comparisons (Table S5). Comparative transcriptomics identified 2246 differentially expressed genes (DEGs), with 1309 up-regulated and 937 down-regulated in the VP-AsA group versus CK (Figure 9).

### 3.9. GO Enrichment Analysis of Differentially Expressed Genes

**Figure 10:**
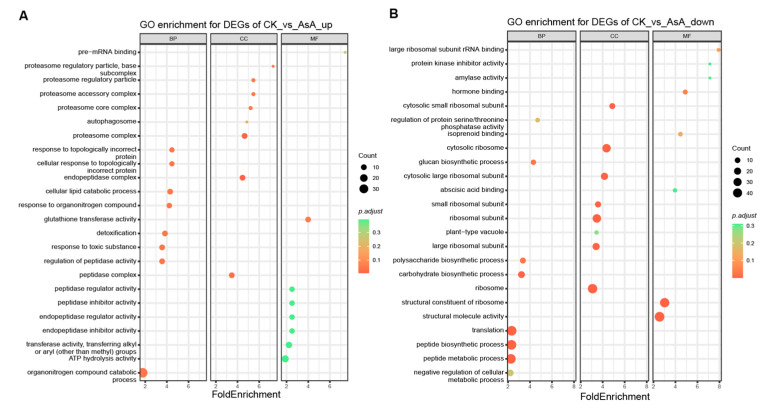
GO enrichment bubble map of differentially expressed genes. Note: BP: biological process; CC: cellular component; MF: molecular function. The color scale on the right represents adjusted *p*-values (p.adjust), while bubble size indicates the number of genes enriched in each term. (**A**) GO enrichment of up-regulated DEGs; (**B**) GO enrichment of down-regulated DEGs.

Functional classification of the annotated differentially expressed genes (DEGs) revealed significant enrichment of Gene Ontology (GO) terms, based on the Solanum tuberosum annotation dataset obtained via Ensembl Plants. Enriched categories spanned the biological process, cellular component, and molecular function domains. As shown in Figure 10, up-regulated DEGs in the VP-AsA group were predominantly enriched in biological processes (e.g., cellular lipid catabolic process, response to organic nitrogen compounds) and cellular components (e.g., proteasome complex), with only glutathione transferase activity significantly enriched in molecular function (Figure 10A). In contrast, down-regulated DEGs were mainly associated with biological processes such as polysaccharide biosynthesis and translation, and cellular components including cytosolic ribosomes, while molecular function terms were limited to structural constituents of ribosomes (Figure 10B). This GO functional enrichment profile highlights key biological processes involved in the VP-AsA–mediated antioxidant response.

### 3.10. KEGG Enrichment Analysis of Differentially Expressed Genes

**Figure 11:**
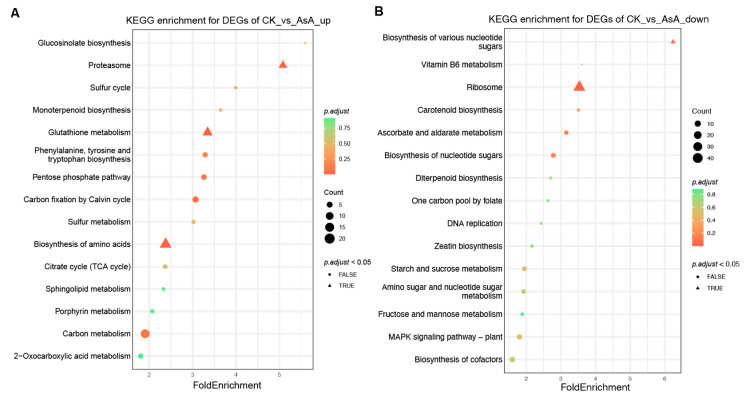
KEGG enrichment bubble map of differentially expressed genes. Note: Bubble size indicates the number of genes enriched in each KEGG pathway (Count). Bubble color represents the adjusted *p*-value (p.adjust), transitioning from green to red as p.adjust decreases. Terms with p.adjust < 0.05 are shown as triangles. (**A**) KEGG enrichment of up-regulated DEGs; (**B**) KEGG enrichment of down-regulated DEGs.

KEGG enrichment analysis identified the primary metabolic and signal transduction pathways associated with the differentially expressed genes (DEGs). As shown in Figure 11, up-regulated DEGs were significantly enriched in the proteasome, glutathione metabolism, and amino acid biosynthesis pathways (Figure 11A), whereas down-regulated DEGs were primarily involved in ribosomal biosynthesis (Figure 11B). These results indicate that VP-AsA treatment predominantly up-regulates antioxidant-related metabolic pathways in fresh-cut potatoes.

### 3.11. Analysis of Differential Genes in Glutathione Metabolism

**Figure 12:**
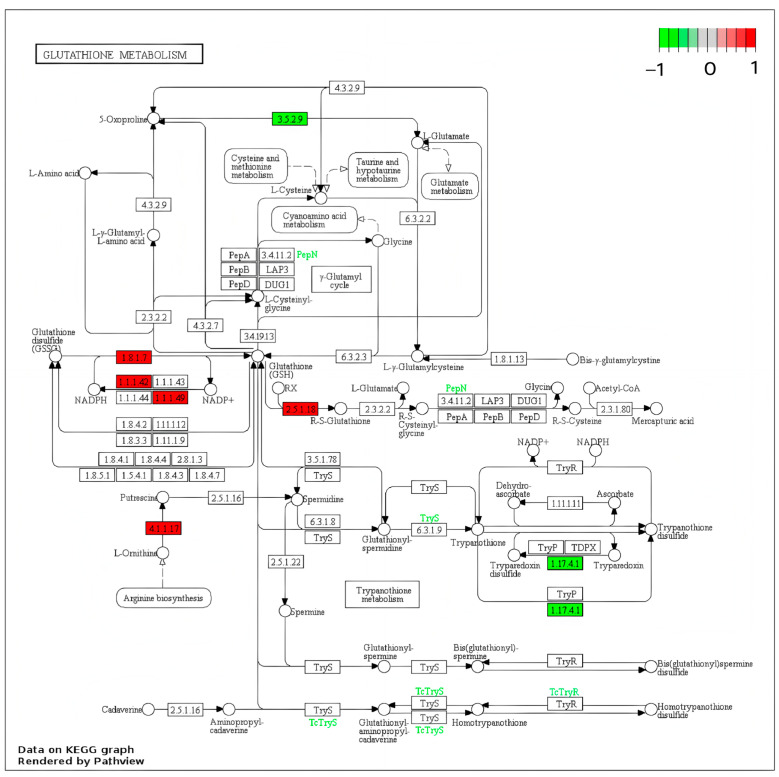
Differentially expressed genes involved in the glutathione metabolism pathway in VP-AsA–treated fresh-cut potatoes at 9 days. Note: Seven key DEGs were highlighted in the pathway: including Glutathione reductase (NADPH) [EC:1.8.1.7], Isocitrate dehydrogenase [EC:1.1.1.42], Glucose-6-phosphate 1-dehydrogenase [EC:1.1.1.49], Glutathione S-transferase [EC:2.5.1.18], Ornithine decarboxylase [EC:4.1.1.17], 5-oxoprolinase (ATP-hydrolyzing) [EC:3.5.2.9] and Ribonucleoside-diphosphate reductase subunit M1 [EC:1.17.4.1]. DEGs: differentially expressed genes; CK: control group; VP-AsA: vacuum packaging combined with 0.5% (*w*/*v*) ascorbic acid treatment.

A detailed analysis of differentially expressed genes (DEGs) within the glutathione metabolism pathway under VP-AsA treatment was conducted. As illustrated in Figure 12, the distribution of DEGs in the glutathione metabolic pathway for the comparison between CK and VP-AsA groups at day 9 of storage revealed significant alterations. Specifically, five key enzyme-encoding genes were significantly up-regulated in response to VP-AsA treatment, including Glutathione reductase (NADPH), Isocitrate dehydrogenase, Glucose-6-phosphate 1-dehydrogenas, Glutathione S-transferase, and Ornithine decarboxylase. Conversely, the expression of the following genes was significantly down-regulated: 5-oxoprolinase (ATP-hydrolyzing) and Ribonucleoside-diphosphate reductase subunit M1.

### 3.12. Proposed Mechanism for Quality Preservation

**Figure 13:**
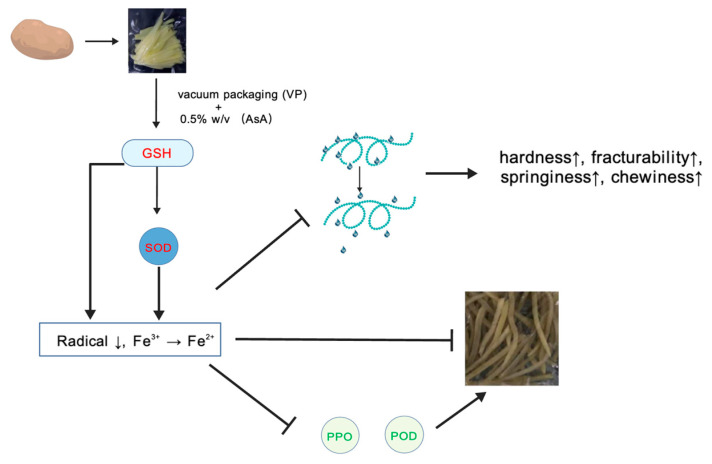
Schematic diagram illustrating the synergistic mechanism of combined vacuum and ascorbic acid treatment (VP-AsA) in maintaining quality of fresh-cut potatoes. In the schematic, upward arrows (↑) indicate promotion or enhancement, while downward arrows (↓) represent inhibition or reduction.

Based on the transcriptomic and physiological findings presented above, a synergistic mechanism of VP-AsA treatment is proposed and summarized in Figure 13; this schematic illustrates that VP-AsA treatment preserves quality through transcriptional regulation that enhances (1) the glutathione-mediated antioxidant system, (2) inhibition of PPO/POD activities, and (3) maintenance of cellular structure integrity, collectively contributing to (4) the preservation of texture and overall quality. These results suggest that VP-AsA treatment preserves quality at least in part by transcriptionally activating glutathione-mediated antioxidant pathways.

## 4. Discussion

This study provides comprehensive insights into the physiological and molecular mechanisms through which combined vacuum packaging and ascorbic acid treatment (VP-AsA) maintains the quality of fresh-cut potatoes during storage. Our findings demonstrate that VP-AsA treatment effectively modulates water migration, preserves textural properties, and enhances antioxidant defense systems.

### 4.1. Water Migration and Distribution Dynamics

The redistribution of water from bound water to free states is a key factor in the quality deterioration of fresh-cut produce. During storage, the observed decrease in bound water content, reflected by increased T_21_ values (indicating enhanced mobility) and a reduced peak area proportion (confirming state transition). This shift may be attributed to cell wall alterations and starch hydrolysis in potatoes, which drive the migration of bound water toward free states due to gradients in water chemical potential.

Wang et al. [35] demonstrated that postharvest pectin degradation in kiwifruit affects hydroxyl protons, leading to increased T_2_ relaxation times. We therefore speculate that the increased T_21_ values during storage of fresh-cut potatoes may be associated with cellular pectin degradation. The VP-AsA treatment effectively reduced pectin degradation and maintained cellular homeostasis. Raffo et al. [36] reported that starch granule hydrolysis during banana ripening increases the shortest relaxation time T_21_ values, while increased sugar concentrations in later storage stages affect water molecule migration rates. These findings may explain the elevated T_21_ values in VP-AsA-treated fresh-cut potatoes at day 15 of storage.

The free water content initially increased then decreased during storage, possibly due to gradual membrane disintegration in potato cells that increased membrane permeability [37]. Additionally, respiratory metabolism in fresh-cut potatoes may generate CO_2_ and water, contributing to increased free water content. By day 15, both treatment groups showed substantially decreased T_23_ signal amplitudes, indicating reduced water mobility and migration of free water into tissue structures as quality deteriorated [38]. Notably, the VP-AsA group exhibited a smaller reduction in free water content compared to the CK group, suggesting its regulatory role in water migration.

MRI observations confirmed these water migration and distribution patterns. The CK group showed severe tissue dehydration and accelerated water migration in later storage stages, evidenced by increased proton density. In contrast, the VP-AsA treatment group maintained more stable water states with minimal structural contraction and water mobility reduction, indicating effective preservation of water status and quality maintenance.

### 4.2. Texture Preservation Mechanisms

The textural property analysis demonstrated that the VP-AsA treatment effectively maintained hardness, fracturability, springiness, and chewiness, thereby delaying quality deterioration. Specifically, the VP-AsA treatment induced only a slight increase in hardness during the early storage period, indicating its regulatory role in the wound healing process on the cut potato surface [11], which may be attributed to the suppression of excessive wound periderm formation. As storage progressed, intensified respiration and nutrient consumption induced by cutting collectively led to a gradual decline in hardness. The CK group exhibited significantly (*p* < 0.05) lower hardness than the VP-AsA group after 9 days, demonstrating the treatment’s efficacy in maintaining hardness over the 15-day storage period. In terms of fracturability, the VP-AsA group remained relatively stable during early storage, with its detectable retention extended to 15 days, whereas it was completely lost in the CK group after 9 days, reflecting the superior ability of the VP-AsA treatment to preserve mouthfeel-related textural characteristics. Furthermore, the VP-AsA group consistently exhibited higher springiness throughout storage, with a significantly (*p* < 0.05) slower decline rate than the CK group after 9 days. Ultimately, the VP-AsA group showed a markedly smaller reduction in chewiness compared to the CK group, further confirming its effectiveness in preserving the sensory and textural quality of fresh-cut potatoes.

### 4.3. The Synergistic Mechanism of VP-AsA Treatment in Modulating PPO and POD Activities

Our results demonstrated that VP-AsA treatment consistently suppressed the activities of both PPO and POD throughout the storage period. As a reducing agent, ascorbic acid may compete with phenolic substrates for the catalytic site of PPO and potentially reduce * o *-quinone intermediates back to less colored compounds. The early suppression of PPO activity by VP-AsA was particularly pronounced. Similarly, VP-AsA strongly inhibited POD activity during the initial storage phase (days 3–9). Although POD is known to be involved in browning and cell wall metabolism in plant tissues, the inhibition observed in this study is based solely on enzyme activity assays. The underlying mechanism may involve the ability of ascorbic acid to scavenge peroxides, which are essential for POD activity, though this hypothesis requires further validation. In summary, based on the enzymatic assays conducted, VP-AsA treatment effectively suppressed both PPO and POD activities.

### 4.4. Enhanced Antioxidant Defense Systems

Superoxide dismutase (SOD), as a crucial component of the ROS scavenging system, plays a vital role in antioxidant processes [39]. Throughout the storage period, the VP-AsA group maintained higher SOD activity than the control, indicating that 0.5% (*w*/*v*) AsA supplementation enhanced antioxidant capacity through increased SOD activity, consistent with previous reports [40].

Plants mitigate reactive oxygen species (ROS) through enzymatic and non-enzymatic antioxidant systems. The key non-enzymatic pathway is the ascorbate-glutathione (AsA-GSH) cycle, in which ascorbate (AsA), glutathione (GSH), and associated enzymes—including ascorbate peroxidase (APX), glutathione reductase (GR), dehydroascorbate reductase (DHAR), and monodehydroascorbate reductase (MDHAR)—cooperate to scavenge excess ROS [41].

**Figure 14:**
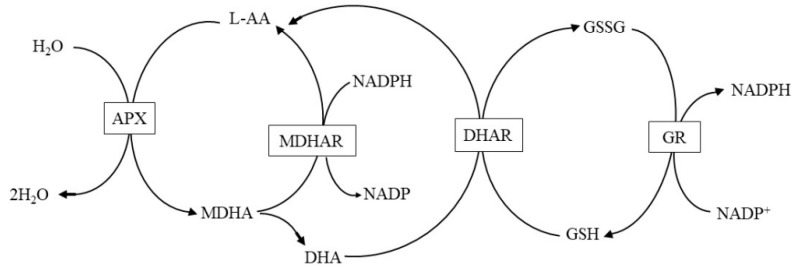
The ascorbate-glutathione cycle [48]. Note: APX: Ascorbate Peroxidase; MDHAR: Monodehydroascorbate Reductase; DHAR: Dehydroascorbate Reductase; GR: Glutathione Reductase; L-AA: L-Ascorbic Acid (Reduced Ascorbate); MDHA: Monodehydroascorbate (Ascorbate Free Radical); DHA: Dehydroascorbate (Oxidized Ascorbate); GSH: Reduced Glutathione; GSSG: Oxidized Glutathione (Glutathione Disulfide); NADPH: Nicotinamide Adenine Dinucleotide Phosphate (Reduced Form); NADP+: Nicotinamide Adenine Dinucleotide Phosphate (Oxidized Form); H_2_O_2_: Hydrogen Peroxide; H_2_O: Water.

Glutathione (GSH), a highly reactive tripeptide, exists in reduced (GSH) and oxidized (GSSG) forms. Reduced GSH acts as a central antioxidant due to its reactive thiol group [42,43]. As depicted in Figure 14, AsA is readily oxidized to monodehydroascorbate (MDHA) and dehydroascorbate (DHA). Unstable MDHA is either reduced back to AsA by MDHAR or disproportionates into AsA and DHA. DHA is then reduced to AsA by DHAR, using GSH as an electron donor and concurrently producing GSSG [44].

Subsequently, GR regenerates GSH from GSSG using NADPH [45]. GSH can also directly reduce H_2_O_2_ to water via enzymes like APX, again yielding GSSG. Furthermore, AsA serves as a key precursor for GSH synthesis, helps maintain thiol reduction, and promotes GSSG-to-GSH conversion. Elevated GSH levels enhance the activities of APX, catalase (CAT), and superoxide dismutase (SOD), fostering further AsA accumulation and establishing a positive feedback loop that collectively boosts the plant’s antioxidant capacity during storage [46,47].

### 4.5. Principal Component Analysis (PCA) of Quality Indicators in Fresh-Cut Potatoes Treated with CK and VP-AsA During Storage

**Figure 15:**
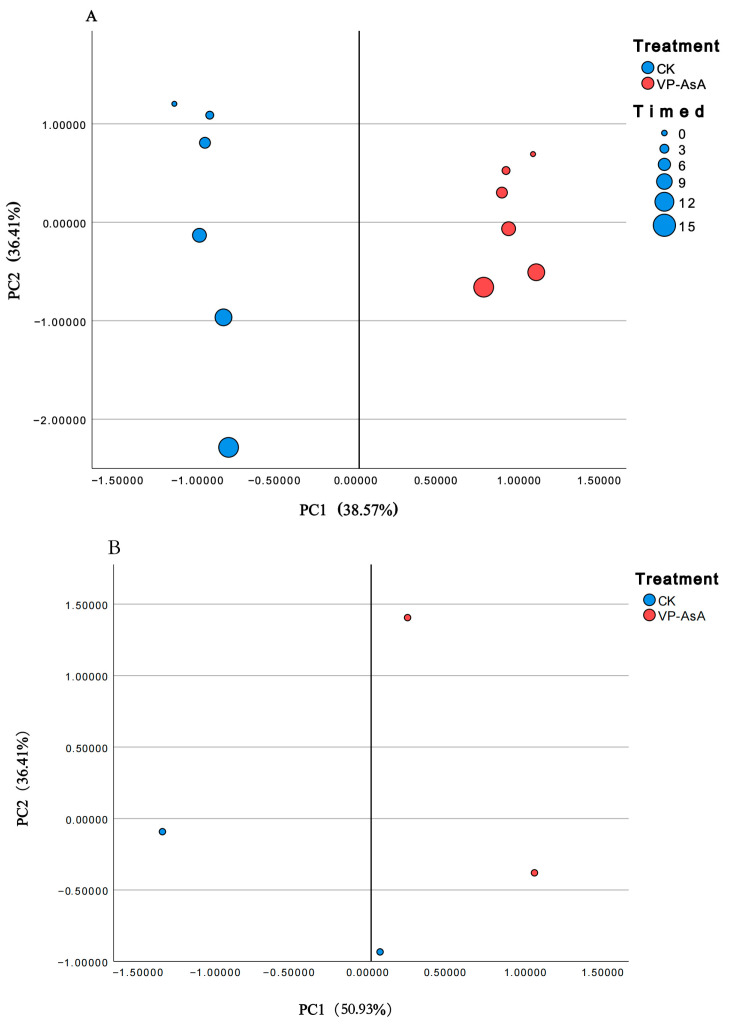
Principal component analysis (PCA) of quality indicators in fresh-cut potatoes treated with CK and VP-AsA during storage. Note: (**A**) PCA score plot across different storage days (excluding transcriptomic indicators); (**B**) PCA biplot of various indicators including transcriptomic data on day 9, the two blue and red dots represent the two biological replicates for the control (CK) and VP-AsA treatment groups on day 9 of storage.

Principal component analysis (PCA) was performed on multiple parameters—including T_21_, T_23_, hardness, fracturability, springiness, chewiness, SOD activities, PPO activities, and POD activities, antioxidant indicators, and GSH content—measured over 0–15 days of storage to comprehensively evaluate the effect of VP-AsA treatment As shown in Figure 15A, the cumulative contribution rate of PC1 and PC2 reached 74.98%, effectively capturing the original data variation. Sample points were distributed across quadrants according to treatment (CK vs. VP-AsA) and storage time. Notably, a distinct separation between the two groups was observed on day 9, indicating fundamental differences in physiological properties such as water status, cellular texture, oxidation, and browning.

To further explore the molecular basis of the physiological divergence observed on day 9, we integrated expression data of five significantly upregulated key enzyme genes with physiological indicators into a subsequent PCA. As presented in Figure 15B, VP-AsA samples shifted positively along the PC1 axis, forming a cluster clearly separated from the CK group. This strongly suggests that VP-AsA treatment induces widespread reprogramming of the gene expression profile, which in turn drives the observed physiological differences.

### 4.6. Transcriptional Regulation of Antioxidant Pathways

Transcriptome analysis further elucidated the molecular mechanisms underlying the enhanced antioxidant activity in VP-AsA-treated fresh-cut potatoes. Significant upregulation of the glutathione metabolism pathway, particularly enhanced glutathione reductase (GSR) activity, indicated increased glutathione recycling that maintained sufficient GSH levels and strengthened antioxidant defense capabilities. The upregulation of GSR and other NADPH-generating enzymes (e.g., isocitrate dehydrogenase and glucose-6-phosphate dehydrogenase) demonstrated reinforced antioxidant defense mechanisms under oxidative stress conditions, increasing NADPH production to support GSH regeneration.

Furthermore, upregulation of glutathione S-transferase suggested involvement in cellular detoxification and antioxidant responses, potentially neutralizing toxic compounds through glutathione conjugation. However, downregulation of 5-oxoprolinase indicated possible reduction in glutathione resynthesis pathways, suggesting potential reliance on existing glutathione recycling rather than de novo synthesis for maintaining antioxidant levels. Downregulation of ribonucleoside-diphosphate reductase subunit M1 implied reduced DNA synthesis and cell proliferation activities, potentially redirecting resources toward antioxidant responses against external stress and oxidative damage. Additionally, AsA itself, as a natural antioxidant, provided direct protective effects against oxidation in fresh-cut potato samples.

This comprehensive analysis demonstrates that VP-AsA treatment improves key quality parameters in fresh-cut potatoes—specifically water status stability, texture, and antioxidant capacity—through mechanisms validated at physiological and molecular levels.

### 4.7. Practical Implications and Industrial Applications

Vacuum-packaged fresh-cut potatoes are pre-processed ingredients in catering. VP-AsA treatment maintained textural properties effectively. These include hardness, fracturability, and chewiness. These traits are critical for thermal processing like frying, baking, and boiling.

AsA at 0.5% (*w*/*v*) is a GRAS food additive. It introduced no noticeable off-flavors. However, its specific flavor impact requires further sensory evaluation.

VP-AsA treatment maintained key measured parameters (texture properties, water status stability, and antioxidant capacity) over 9 days. This provides a foundation for potential shelf-life extension to 15 days. Actual shelf-life must be determined via microbial safety and sensory acceptance thresholds.

It is important to note the study’s limitations. These findings are from a lab-scale study using one potato cultivar. Generalizability needs validation across other varieties and real supply chain conditions. Future work must include sensory evaluation, microbial safety assessments, and commercial-scale testing. These steps are essential for practical application.

## 5. Conclusions

This study demonstrates that combined vacuum packaging and 0.5% (*w*/*v*) ascorbic acid treatment (VP-AsA) mitigates quality deterioration in fresh-cut potatoes through multiple mechanisms (Figure 15). The VP-AsA treatment modulated water status by retarding the migration of bound water to free water, contributing to cellular water homeostasis. It effectively suppressed the activities of polyphenol oxidase and peroxidase and better maintained textural properties including hardness and fracturability, thereby delaying browning and texture softening. At the molecular level, the treatment enhanced the antioxidant defense system by activating the glutathione metabolism pathway, where the upregulation of key genes such as glutathione reductase indicates improved oxidative stress tolerance. Taken together, this work provides the first evidence that the efficacy of VP-AsA is fundamentally linked to its transcriptional regulation of glutathione metabolism. Thus, it proposes a practical, low-additive strategy to extend the shelf-life of minimally processed vegetables. More broadly, these findings underscore the potential of applying transcriptome analysis to develop targeted interventions for fresh-cut fruits and vegetables quality control.

## Data Availability

The original contributions presented in this study are included in the article/Supplementary Materials. Further inquiries can be directed to the corresponding author.

## Supplementary Materials

The following supporting information can be downloaded at: https://www.mdpi.com/article/10.3390/foods15010035/s1, Table S1. Changes in water content of fresh-cut potatoes during storage. Table S2. Effects of VP-AsA treatment on DPPH radical scavenging activity (%). Table S3. Effects of VP-AsA treatment on ABTS radical scavenging activity (%). Table S4. Effects of VP-AsA treatment on FRAP. Table S5. Summary of transcriptome sequencing data quality for all samples.
