# Supplementary material for: Combined Vacuum and Ascorbic Acid Treatment Enhances Texture and Antioxidant Capacity in Fresh-Cut Potatoes: Transcriptomic Elucidation of Glutathione Metabolism Mechanisms
Source: Foods. 2025 Dec 22;15(1):35. doi: 10.3390/foods15010035 (PMC12786257; doi:10.3390/foods15010035)
Supplement: Supplementary file 1 [file foods-15-00035-s001.zip › foods-4018975-supplementary.pdf]

Supplementary Material

Table S1. Changes in water content of fresh-cut potatoes during storage.

Treament	Storage time/d	A_{21}(%)	A_{23}(%)	（A_2）
CK	0	7.51+0.71^{a}	91.99+0.62^{abc}	2111.30±134.72^{c}
	3	6.70+0.25^{abcd}	93.44+0.30^{ab}	2494.60±184.24^{ab}
	6	6.22+0.37^{cd}	93.17+1.10^{ab}	2150.52±22.57^{c}
	9	6.02+0.21^{de}	94.10+0.21^{a}	2159.68±54.32^{bc}
	12	5.21+0.94^{ef}	93.42+0.62^{ab}	2305.42±54.24^{abc}
	15	3.45+0.92^{g}	88.57+0.62^{d}	1606.20±8.18^{d}
VP-AsA	0	7.26+1.28^{ab}	90.86+0.62^{cd}	2144.94±78.06^{c}
	3	7.23+0.25^{ab}	93.27+0.88^{ab}	2527.89±96.97^{a}
	6	6.97+0.15^{abc}	92.97+0.13^{abc}	2129.19±86.33^{c}
	9	6.49+0.37^{bcd}	93.51+0.37^{ab}	2216.98±144.48^{abc}
	12	6.07+0.27^{cde}	93.90+1.87^{a}	2235.66±75.46^{abc}
	15	5.01+0.09^{f}	91.75+1.34^{bcd}	1737.73±63.16^{d}

Note: Different letters within the same column indicate significant differences at ($P < 0.05$).

Table S2 Effects of VP-AsA treatment on DPPH radical scavenging activity (%)

Source	Mean Square	F	P-value
Corrected Model	1309.502	54.292	0.000
Intercept	145547.337	6034.441	0.000
Time (d) (CK, VP-As A)	28.743	1.192	0.343
Treatment	14144.345	586.429	0.000
Time (d) * Treatment	23.292	0.966	0.458
Error	24.119		

Table S3 Effects of VP-AsA treatment on ABTS radical scavenging activity (%)

Source	Mean Square	F	P-value
Corrected Model	88.41	65.11	0.000
Intercept	6009.867	4425.972	0.000
Time (d) (CK, VP-As A)	5.506	4.055	0.008
Treatment	910.631	670.635	0.000
Time (d) * Treatment	6.87	5.059	0.003
Error	1.358		

Table S4 Effects of VP-AsA treatment on FRAP

Source	Mean Square	F	P-value
Corrected Model	0.045	89.742	0.000
Intercept	11.719	23308.928	0.000
Time (d) (CK, VP-As A)	0.003	5.101	0.003
Treatment	0.427	848.972	0.000
Time (d) * Treatment	0.011	22.537	0.000
Error	0.001		

Table S5. Summary of transcriptome sequencing data quality for all samples

Sample	Raw Reads	Raw Bases	Clean Reads	Clean Bases	Error Rate (%)	Q20 (%)	Q30 (%)	GC Content (%)
CK-1	42,677,880	6.41 Gb	42,446,204	6.41 Gb	0.03	97.74	93.42	42.63
CK-2	39,266,396	5.90 Gb	39,033,586	5.89 Gb	0.03	97.91	93.83	42.65
AsA-1	39,045,442	5.87 Gb	38,849,224	5.87 Gb	0.03	97.76	93.43	42.7
AsA-2	42,810,602	6.44 Gb	42,610,774	6.43 Gb	0.03	97.56	93.00	42.64
